# Home and Work Physical Activity Environments: Associations with Cardiorespiratory Fitness and Physical Activity Level in French Women

**DOI:** 10.3390/ijerph13080824

**Published:** 2016-08-15

**Authors:** Jean-Michel Oppert, Marie-Aline Charles, Hélène Charreire, Mehdi Menai, Ilse De Bourdeaudhuij, Soren Brage, Blandine de Lauzon-Guillain, Guy Fagherazzi, Beverley Balkau

**Affiliations:** 1 Department of Nutrition Pitié-Salpêtrière Hospital, Institute of Cardiometabolism and Nutrition, Assistance Publique-Hôpitaux de Paris, Sorbonne Universités, Université Pierre et Marie Curie, Université Paris 06, Paris F-75013, France; 2 Nutritional Epidemiology Research Team (EREN), Sorbonne Paris Cité Epidemiology and Statistics Research Center, Inserm U1153, Inra U1125, Conservatoire National des Arts et Métiers (CNAM), University of Paris 13, Bobigny F-93017, France; menai.mehdi0@gmail.com; 3 Early Origin of the Child Health and Development Team (ORCHAD), INSERM, UMR1153 Epidemiology and Biostatistics Sorbonne Paris Cité Research Center (CRESS), Paris Descartes University France, Paris F-75014, France; marie-aline.charles@inserm.fr (M.-A.C.); blandine.delauzon@inserm.fr (B.L.-G.); 4 Lab-Urba Urbanism Institute of Paris, University of Paris Est, UPEC, Créteil F-94010, France; helene.charreire@u-pec.fr; 5 Department of Movement and Sports Sciences, Faculty of Medicine and Health Sciences, Ghent University, Watersportlaan 2, Ghent 9000, Belgium; ilse.debourdeaudhuij@UGent.be; 6 MRC Epidemiology Unit, Institute of Metabolic Science, University of Cambridge, Box 285, Addenbrookes Hospital, Hills Road, Cambridge CB2 0QQ, UK; soren.brage@mrc-epid.cam.ac.uk; 7 INSERM UMR-S 1018 (CESP), Universities Paris South and St Quentin-en-Yvelines, Villejuif Cedex F-94807, France; guy.fagherazzi@gustaveroussy.fr (G.F.); beverley.balkau@inserm.fr (B.B.)

**Keywords:** home environment, workplace environment, physical activity, physical fitness, aerobic capacity, accelerometer, heart rate, questionnaire

## Abstract

The influence of the physical activity environment in the home and at work on cardiorespiratory fitness (CRF) and objectively-measured physical activity has not been extensively studied. We recruited 147 women with a (mean ± SD) age of 54 ± 7 years and without evidence of chronic disease. The physical activity environment was assessed by self-report (Assessing Levels of PHysical Activity or ALPHA questionnaire), CRF using a submaximal step test, usual physical activity using combined heart rate and accelerometry, as well as by a validated questionnaire (Recent Physical Activity Questionnaire). Summary scores of the home environment and the work environment derived from the ALPHA questionnaire were positively correlated with CRF after adjustment for age (*r* = 0.18, *p* = 0.03 and *r* = 0.28, *p* < 0.01, respectively). Women owning a bicycle or having a garden (which may prompt physical activity) had higher CRF; those with a bicycle at home also had a higher physical activity energy expenditure. Similarly, women who had access to fitness equipment at work had higher CRF. In conclusion, these results provide new insights into potential environmental influences on physical capacity and physical activity that could inform the design of physical activity promotion strategies.

## 1. Introduction

The promotion of physical activity (PA) is recognized as a major component in the prevention of non-communicable diseases [1]. Designing effective PA interventions requires prior knowledge of correlates of usual PA at the population level [2]. Over the last decade, the development of socio-ecological models of health behaviors has focused attention on the potential influence of environmental factors—both physical and social—on usual PA [3]. Characteristics of the built environment, such as residential density, density of destinations, availability of sidewalks, cycle paths and recreational facilities, as well as neighborhood safety, have been associated with a physically active lifestyle [4]. However, environmental settings of interest include not only the residential neighborhood (e.g., recreation and transportation facilities, land use, etc.) but also the home and workplace PA-related environments.

There is evidence of an association between indoor PA or exercise equipment in the home and usual PA levels, as recently reviewed [5]. Studies in adults are much fewer than in youth [6]. Based on self-reported PA, summary scores of home PA equipment were found to be associated with the adoption or practice of PA in American [7,8,9,10,11,12], Belgian [13,14], and Japanese [15] adults. Similar associations were found with accelerometer-measured moderate-to-vigorous PA in one study [14], but not another [9]. In most studies, only summary scores of home PA equipment are presented, but a precise assessment of the potential benefit of each component of the PA environment available is needed. In addition, despite documented positive effects of PA interventions at the workplace on PA and physical fitness, better knowledge of the associations between PA equipment or fitness classes provided at work and the level of PA of workers is needed [16].

Physical fitness and PA are two different, although related, concepts [17]. PA is a multidimensional and complex behavior, thus difficult to measure. However, recent technological developments have made it feasible to obtain objective assessments of PA in everyday-life conditions, in medium-to-large-scale studies, using accelerometry and heart rate recordings [18]. On the other hand, cardiorespiratory fitness (CRF) or cardiorespiratory endurance is a capacity measure; CRF is less prone to intra-individual day-to-day variability than PA, and is generally measured with more accuracy [17]. CRF can be estimated from heart rate response to submaximal exercise or exercise time to exhaustion during a maximal test. Low CRF is recognized as a major risk factor for all-cause mortality and cardiovascular events [17]; it is also a marker of functional capacity and ability to perform the tasks of daily living adequately. CRF depends, in part, on the usual level of PA, but CRF and PA are independently associated with health outcomes such as cardiometabolic risk factors [19]. To our knowledge, the influence of PA equipment at home or work (which may prompt PA behavior) on CRF has not been extensively studied. 

This study analyzes the relationships between characteristics of the PA-related home and work environment obtained by questionnaire with CRF and PA. Findings show that summary scores of the home environment (e.g., possessing exercise equipment) and of the workplace environment (e.g., availability of equipment or sports clubs) are positively correlated with CRF.

## 2. Materials and Methods

### 2.1. Study Design and Participants

This study was part of the EU-funded EPIC-InterAct project [20]. As part of this project, a specific study was set up to validate the EPIC PA questionnaire used in participating countries by using objective measures of PA [21]. Results of this validation study were reported elsewhere [21]. The women recruited for the validation study were selected to mirror the participants in the original EPIC (European Prospective Investigation into Cancer and Nutrition) study. In France, the EPIC study is based on the “Etude Epidémiologique auprès des Femmes de la Mutuelle Générale de l'Education Nationale” (E3N) cohort, an ongoing cohort including women who are covered by the French national teachers’ insurance plan [22]. To be included in the present study, women had to be between the ages of 30 and 65 years, to live in the Île-de-France region that includes the city of Paris and its suburbs, and to be free of cardiovascular disease (angina pectoris, myocardial infarction, heart failure, cardiomyopathy, stroke, peripheral arterial disease), respiratory diseases and diabetes, as assessed during an examination by a physician. In addition, they were not pregnant, did not use beta blockers, and did not have any physical disability preventing them from walking unaided for a minimum of 10 min; all women completed a screening questionnaire on the safety of exercising. 

Women were invited to two sessions, three months apart, at the Centre for Research on Human Nutrition Île-de-France (CRNH IdF) at the Pitie-Salpêtrière Hospital (Assistance Publique-Hôpitaux de Paris, Paris, France). This design was followed to be able to better capture physical activity patterns in free living conditions. At the first session, the study was explained. At both sessions, anthropometric measures were recorded, and a step test was carried out for the assessment of CRF and individual heart rate calibration. Women were then asked to wear an activity monitor for at least four days, to be returned by post. At the second session, they completed questionnaires on their usual PA, and also the Assessing Levels of Physical Activity (ALPHA) questionnaire (see below). The study protocol was approved by the ethics committee of the Kremlin-Bicêtre hospital (Paris, France) (number CPP 08-018), and the Institut National de la Santé et de la Recherche Médicale (INSERM) was the guarantor for the study. All women signed an informed consent.

In total, 175 women were recruited. Six women were excluded from the present analyses because of insufficient PA data, and a further 22 were not included because they did not complete the ALPHA questionnaire or lived outside the region. The present analyses therefore include 147 women, except for the analysis concerning the workplace environment, where we studied the women who reported that they worked (*n* = 105).

### 2.2. Cardiorespiratory Fitness Measurement

A validated combined heart rate and movement sensor (Actiheart, CamNtech, Cambridge, UK) was fitted on the chest of the participating women [23]. The Actiheart is a small (33 mm diameter × 7 mm thick), light weight (<8 g), waterproof monitor which simultaneously measures heart rate (128 Hz) and uniaxial acceleration (32 Hz) by a piezoelectric element [23]. During the step test, subjects also wore a separate heart rate monitor with a display (Polar FS2C, POL90027148). An 8 min sub-maximal ramped step test (200 mm step; Reebok, Lancaster, UK) was performed, followed by a 2 min recovery phase. Heart rate was recorded throughout the exercise test and recovery phase. Based on the data on heart rate and work load, CRF (VO_2max_ (mL/kg/min)) was estimated by extrapolating the individually established relationship between oxygen consumption and heart rate [24] to age-predicted maximal heart rate [25]. The average of two estimated VO_2max_ values (from the step test performed at each of the two study visits), weighted by test duration, was used in the analyses.

### 2.3. Objective Physical Activity Measurement

Following the step test, the combined sensor was initialized for long-term recording of PA in 1 min epochs. Participants were instructed to wear the monitor continuously for a minimum of four days, while doing their normal daily activities. Activity intensity (J/kg/min) was estimated for each time point, from the combination of torso acceleration and individually calibrated heart rate, using a branched equation framework, as previously described [24]. Next, non-wear periods were identified from the combination of non-physiological heart rate and prolonged periods of inactivity. We accounted for any potential diurnal imbalance of wear time by weighting all hours of the day equally in the summation [26]. The intensity time-series was summarized into total PA energy expenditure (PAEE, kJ/kg/day) [27] and time (% day) spent at a PA intensity of 0 J/min/kg, representing time spent in sedentary pursuits including sleep. Estimated PAEE from this method compares favorably to PAEE measured by doubly-labelled water (no mean bias and correlation of *r* = 0.66) [27]. Two Actiheart recordings were available in all but nine women who only had one record; the mean value was used when available.

### 2.4. Physical Activity Questionnaire

At the second visit, participants completed the Recent Physical Activity Questionnaire (RPAQ) [28]. The RPAQ is designed to assess usual PA in the last four weeks, and measures of PAEE and daily time spent sedentary can be estimated. Details about questionnaire calculations are provided elsewhere [28]. A validation study of RPAQ using a sample of adults of working age has shown the estimated total PAEE to have good test-retest reliability and criterion validity against measured PAEE, when compared to other self-reported instruments described in the literature [28]. Intraclass correlation coefficients (ICC) for two-week test-retest repeatability were 0.76 (*p* < 0.001) for both PAEE and time spent sedentary [28]. Estimated PAEE was significantly associated with PAEE measured over 14 days by doubly-labelled water (*r* = 0.39, *p* = 0.0004; mean difference ± SD = −12.9 ± 23.9 kJ/day, *p* < 0.05) [28].

### 2.5. Physical Activity Environment Questionnaire

At the second visit, participants also completed the ALPHA questionnaire [29,30]. This self-administered instrument was designed in the framework of the EU-funded project “Instruments for Assessing Levels of PHysical Activity and fitness” to assess perceptions about characteristics of the physical environment related to PA behavior [29]. In a pilot study, the questionnaire was found to have good reliability and predictive validity (regarding context-related PA) in selected population samples from Austria, Belgium, France, and the UK [30]. ICCs for one-week test-retest reproducibility of ALPHA environmental scores were shown to range from 0.66 to 0.87, and significant associations were found between ALPHA environmental scales and accelerometry-measured physical activity levels, particularly in women [30]. The questionnaire covers nine themes, including home environment (six items) and work (or study) environment (11 items). These items were scored according to a two-point scale (presence: yes-no). Scoring of items from the ALPHA questionnaire was processed according to previously reported procedures [29].

### 2.6. Other Measures

Weight and height were measured by a standard scale (Seca scales, 761, Seca, Birmingham, UK) and a rigid and portable stadiometer (Leicester stadiometer, Seca, Birmingham, UK), respectively. Body mass index (BMI) was calculated as weight (kg)/height^2^ (m). As anthropometry was assessed at both time points, all measures used in the analysis were averaged.

### 2.7. Statistical Analysis

Continuous variables are characterized by means and standard deviations (SD), and categorical variables by percentages. We constructed scores of the PA-related environment in the home and at work as the sum of the presence of each item of the environment, according to the ALPHA protocol [29]. Analysis of covariance (ANCOVA) with adjustment for age was used for the comparison of PA and CRF measures according to answers (yes-no) from the ALPHA questionnaire related to the home and work environments. Similar results were obtained using non-parametric tests for the continuous variables. Relationships between the ALPHA questionnaire scores and PA and CRF measures were assessed using Spearman correlation coefficients with adjustment for age. Statistical significance was set at *p* < 0.05. All analyses used SAS software (SAS, version 9.3, SAS Institute Inc., Cary, NC, USA). 

## 3. Results

Table 1 shows the characteristics of the women studied, and the CRF and PA data obtained from combined sensing and self-report. PAEE was 37.1 kJ/kg/day by the objective measure and 33.2 kJ/kg/day by self-reported measure. From the 6-and 10-point scale for home and work PA-related environments, respectively, participants reported a mean score of 1.9 for home and 4.2 for work. For some items (e.g., “Bicycles provided” at work), the number of positive responses was low). 

Table 2 shows the relationships between the ALPHA PA-related home and work scores with CRF and PA data obtained from both objective and self-report measures. Both home and work PA-related environment scores were positively correlated with CRF (*r* = 0.18 and *r* = 0.28, respectively). 

Table 3 shows mean values of CRF, PAEE, and time spent sedentary, according to items related to the home and work environments from the ALPHA questionnaire. For the home environment items, women with their own bicycle and those with a garden in their home had a significantly higher CRF; women with a personal bicycle also had a higher PAEE and a shorter time spent sedentary (by RPAQ-reported methods). Those who had access to a car also had higher PAEE and tended to have less sedentary time. For the work environment items, less consistent patterns of association were found. Women who had fitness equipment provided at work had a significantly higher CRF.

## 4. Discussion

In this study, we assessed the relationships between characteristics of the self-reported home and work environments related to PA and actual physical fitness and PA levels in French women. The home and work environments were assessed by the ALPHA self-reported questionnaire, specifically designed for European studies. Usual PA was measured by both objective and subjective methods. A main result was that some home, and, to a lesser extent, some workplace characteristics were significantly related with CRF in these women.

Although data on the population distribution of fitness levels are limited, CRF levels in our population appear to be similar to previously-reported descriptive data. In the 1999–2004 NHANES survey, the median maximal oxygen uptake estimated from a submaximal treadmill test was 33.3 mL/kg/min in women aged 40–49 years [31] compared to 31.7 mL/kg/min in our women aged 54 years on average. We report significant relationships between attributes of the self-reported indoor PA-related environment and a measure of CRF in adult women. The data suggest the importance of the availability of equipment in the home environment. To have a personal bicycle or a garden at home was positively associated with CRF and usual PA-related energy expenditure. CRF is a predictor of CVD risk and all-cause mortality in women as in men [32].

There are very few published data based on objective fitness assessment with which to compare our findings. Previous studies have documented (in adults) associations between the availability of PA/sporting equipment at home and usual PA—either self-reported [7,8,9,10,11,12,13,14,15] or recorded by accelerometry [14]—in general agreement with our findings. Interestingly, several studies using PA questionnaires showed associations between home PA equipment and self-reported vigorous leisure PA [9,14], a type of PA more likely to be associated with increased CRF than light or moderate-intensity PA. In one Australian study in older adults, no association was found between home PA equipment and self-reported PA [33]. However, in that study, questions on home equipment pertained only to the presence of an exercise bike, a swimming pool, or exercise videos, in contrast to the detailed assessment of home equipment in our study.

In an early 12-month non-controlled PA promotion study in adults, a summary score of home exercise equipment was found more important in predicting PA adoption rather than maintenance [10]. Additional insight is provided by data from controlled intervention studies. Secondary analyses from two individual-level trials that aimed to increase PA among inactive adults by providing motivational material, but did not aim to change environmental perceptions, found that perceived home equipment availability increased in the intervention groups [11]. In addition, home equipment availability was associated with increased PA [11]. Altogether these findings and our data point to a role for the home environment in providing ease of access to a variety of equipment and material which, in turn, could prompt PA behavior [5].

Interestingly, possessing equipment at home (such as a bicycle) was not only associated with increased CRF, but was also associated with less time spent sedentary. Previous studies indicate positive relationships between equipment promoting sedentary time (number of TVs and computers in the home) with time spent in front of a screen, a typical sedentary behavior [34]. Our data suggest that home-based equipment could be inversely associated with sedentary time, a finding which would need to be confirmed in other settings and populations.

Specifically for the women in this study who worked—the presence of fitness equipment at work, was favorably associated with CRF. These findings add to the evidence that a PA-supporting environment in the workplace would encourage PA and increase fitness [16,35]. Access to worksite PA amenities (e.g., pleasant place to walk, fitness facility, showers) was associated with higher leisure-time PA in some studies [36] but not others [37]. Associations in this study of worksite PA-related environment with higher CRF are in line with previous results indicating increased self-reported vigorous PA with opportunities to be active in the worksite environment [38]. The size of our sample is, however, too small to draw solid conclusions.

Among the strengths of the study, the women underwent a submaximal exercise test allowing the assessment of CRF—a recognized predictor of CVD risk and all-cause mortality [17,32]. Another strength is the use of both accelerometry plus heart rate-based and questionnaire-based measures of usual PA, in an attempt to overcome the limitations of both subjective and objective assessments of PA. To measure home and work PA-related environmental factors of interest, we used the ALPHA questionnaire—an instrument specifically designed to assess an individual’s perceptions of the neighborhood-related physical environment in European settings—and it included some indoor characteristics.

Among the study limitations, the data were cross-sectional, therefore preventing the possibility of drawing conclusions about causality or temporality. Women were 54 years old on average and were mainly drawn from a larger population covered by the French national teachers’ insurance plan, which may limit generalizability of the findings. We did not have precise data on professional work performed by subjects, but, given the recruitment strategy, most of them were teachers or researchers. Data about the PA-related home and work environment in this study were subjective, based on perception of the environment as assessed by the ALPHA questionnaire. However, self-reporting of environmental characteristics remains easy and less expensive than other possible options (e.g., field audit by rater at home or at the workplace of each participant). It is anticipated that advances in information technologies will bring new tools for real-time mobile sensing of PA as well as assessment of various settings of interest [39].

## 5. Conclusions

In this study, we have investigated the relationships between home and work PA-related environment and a major health indicator, physical fitness. The results indicate that certain home and workplace characteristics, such as the presence of a bicycle or a garden at home appear to be positively related with higher physical fitness. These findings emphasize the importance of not only focusing on urban form and attributes but also taking into account what is available in the immediate living environment—at home and at work—when designing strategies to promote PA and increase physical fitness in adults residing in European urban settings.

## Figures and Tables

**Table 1:** Characteristics of the 147 French women and measures of cardiorespiratory fitness, physical activity energy expenditure, and home and work (*n* = 105) physical activity-related environment scores.

Individual Characteristics	Mean (SD) or *n* (%)
Age (years)	54 (7)
Weight (kg)	60.7 (8.7)
Height (m)	1.63 (0.01)
BMI (kg/m^2^)	22.9 (3.0)
Objective fitness and activity measures	
CRF (mL O_2_/kg/min) *	31.7 (4.4)
PAEE (kJ/kg/day) *	37.1 (11.9)
Sedentary time (%day) *	50.4 (7.3)
Self-reported activity measures	
PAEE (kJ/kg/d)	33.2 (16.0)
Sedentary time (h/day)	22.2 (2.1)
Physical activity related environment	
*Home score*	1.9 (1.3)
Personal bicycle	75 (51%)
Garden	47 (32%)
Sports equipment, racquets	74 (50%)
Exercise equipment	45 (31%)
Access to car	123 (84%)
Dog	16 (11%)
*Work score (n = 105)*	4.2 (1.8)
Escalators, lifts	76 (72%)
Stairs	99 (94%)
Fitness equipment	30 (29%)
Bicycles provided	3 (3%)
Safe place to leave bike	55 (52%)
Enough car parking	54 (51%)
Showers, change rooms	24 (23%)
Exercise classes	47 (45%)
Sports club	41 (39%)
Subsidized public transport	61 (58%)

CRF: cardiorespiratory fitness; PAEE: Physical activity energy expenditure; * The mean of two observations is used, except for the nine women where there was only one recording.

**Table 2:** Spearman correlation coefficients (*r*) between the home and work physical activity-related environment scores and physical activity measures.

ALPHA Scores	Objective Measures						Self-Reported Measures (RPAQ)			
	CRF (mL O_2_/kg/min)		PAEE (kJ/kg/day)		Sedentary Time (%day)		PAEE (kJ/kg/day)		Sedentary Time (h/day)	
	*r*	*p*	*r*	*p*	*r*	*p*	*r*	*p*	*r*	*p*
Home environment (*n* = 147)	0.18	**0.03**	0.01	0.91	0.01	0.94	0.13	0.12	0.13	0.11
Work environment (*n* = 105)	0.28	**<0.001**	0.13	0.12	0.09	0.29	0.05	0.52	0.10	0.25

ALPHA: Assessing Levels of PHysical Activity questionnaire; CRF: cardiorespiratory fitness; PAEE: Physical activity energy expenditure. *r* are age-adjusted partial correlation coefficients. Significant *p* values are in bold font.

**Table 3:** Mean values of measures of cardiorespiratory fitness and of physical activity by objective and self-reported methods, according to the presence of home (*n* = 147) and work (*n* = 105) physical activity-related environment items.

SCALE, Items	CRF (mL O_2_/kg/min)			Objective PAEE (kJ/kg/day)			Sedentary Time (%/day)			Self-Reported PAEE (kJ/kg/day)			Sedentary Time (h/day)		
	Has Equipment		*p* Value	Has Equipment		*p* Value	Has Equipment		*p* Value	Has Equipment		*p* Value	Has Equipment		*p* Value
	NO	YES		NO	YES		NO	YES		NO	YES		NO	YES	
***Home environment (n = 147)***															
Personal bicycle	30	33	**0.03**	34.7	39.4	0.19	52	49	0.30	27.7	38.5	**<0.01**	22.7	21.8	**0.05**
Garden	31	33	**0.03**	36.1	39.0	0.32	51	50	0.95	33.2	33.2	0.84	22.1	22.5	0.23
Sports equipment, racquets	31	33	0.24	35.7	38.5	0.85	52	49	0.18	30.2	36.1	0.11	22.6	21.9	0.21
Exercise equipment	31	32	0.68	36.1	39.3	0.71	51	49	0.75	31.5	36.9	0.19	22.5	21.6	**0.05**
Access to car	31	32	0.83	33.3	37.8	0.16	52	50	0.22	25.8	34.6	**0.024**	23.0	22.1	0.06
Dog	32	33	0.53	36.8	39.6	0.63	50	51	0.41	33.0	34.8	0.85	22.2	22.9	0.09
***Work environment (n = 105)***															
Escalators, lifts	33	33	0.26	40.5	38.9	0.39	50	49	0.29	32.2	36.1	0.23	22.2	21.8	0.37
Stairs	32	33	0.94	38.5	39.4	0.96	52	49	0.34	37.1	34.9	0.71	21.9	21.9	0.99
Fitness equipment	32	34	**0.05**	39.3	39.5	0.79	49	50	0.23	36.0	32.5	0.26	21.6	22.5	**0.05**
Bicycles provided	33	37	0.06	39.1	46.7	0.26	49	50	0.85	35.2	28.7	0.46	21.9	22.1	0.84
Safe place to leave bike	32	33	0.22	39.2	39.4	0.96	50	49	0.49	33.4	36.5	0.28	21.5	22.2	0.10
Enough car parking	32	33	0.30	39.6	39.1	0.84	49	50	0.85	35.1	35.0	0.97	21.5	22.2	0.11
Showers, change rooms	33	33	0.84	39.3	39.4	0.87	49	48	0.27	33.6	39.7	0.07	22.1	21.1	**0.05**
Exercise classes	32	34	0.15	39.5	39.1	0.54	49	49	0.59	35.4	34.2	0.60	21.4	22.4	**0.02**
Sports club	33	33	0.99	41.1	36.6	**0.03**	48	51	**0.016**	36.7	32.3	0.12	21.4	22.6	**0.01**
Subsidized public transport	32	33	0.09	39.8	39.0	0.52	49	49	0.70	36.6	33.8	0.33	21.7	22.0	0.60

CRF: cardiorespiratory fitness; PAEE: Physical activity energy expenditure. Analyses are age-adjusted. Significant *p* values are in bold font.

## Abbreviations

The following abbreviations are used in this manuscript:

ALPHA	Assessing Levels of Physical Activity and fitness
BMI	body mass index
CRF	cardiorespiratory fitness
CVD	cardiovascular disease
EPIC	European Prospective Investigation into Cancer and Nutrition
E3N	Etude Epidémiologique auprès des Femmes de la Mutuelle Générale de l’Education Nationale
CRNH IdF	Centre for Research on Human Nutrition Île-de-France
INSERM	Institut National de la Santé et de la Recherche Médicale
MET	metabolic equivalent task
NHANES	National Health and Nutrition Examination Survey
PA	physical activity
PAEE	physical activity energy expenditure
r	Spearman correlation coefficient
RPAQ	Recent Physical Activity Questionnaire
